# Effects of Exercise in Patients Undergoing Chemotherapy for Head and Neck Cancer: A Pilot Randomized Controlled Trial

**DOI:** 10.3390/ijerph18031291

**Published:** 2021-02-01

**Authors:** Kuan-Yin Lin, Hui-Ching Cheng, Chia-Jui Yen, Ching-Hsia Hung, Yu-Ting Huang, Hsin-Lun Yang, Wan-Ting Cheng, Kun-Ling Tsai

**Affiliations:** 1 Department of Physical Therapy, College of Medicine, National Cheng Kung University, Tainan 701, Taiwan; 10802003@gs.ncku.edu.tw (K.-Y.L.); qaz200408@gmail.com (H.-C.C.); chhung@mail.ncku.edu.tw (C.-H.H.); 530.yuting@gmail.com (Y.-T.H.); t66064043@pt.ncku.edu.tw (H.-L.Y.); myenthusiasticlife@gmail.com (W.-T.C.); 2 Institute of Allied Health Sciences, College of Medicine, National Cheng Kung University, Tainan 701, Taiwan; 3 Division of Hematology and Oncology, Department of Internal Medicine, Graduate Institute of Clinical Medicine, National Cheng Kung University Hospital, Tainan 701, Taiwan; yencj@mail.ncku.edu.tw

**Keywords:** head and neck cancer, exercise training, muscle strength, chemotherapy

## Abstract

Background: Cisplatin administration may induce muscle atrophy, thereby reducing the fitness level of patients with head and neck cancer (HNC). To date, only animal studies have been conducted to test the effectiveness of exercise interventions in diminishing side effects of cisplatin. Aim: To determine whether exercise training improves physical fitness and health-related quality of life (HRQoL) in patients receiving chemotherapy for Head & Neck (H&N). Material and methods: This pilot-randomized controlled trial was conducted on 57 participants receiving chemotherapy for HNC. The participants were randomized into an exercise group and a control group. The exercise group received moderate-intensity combined aerobic, resistance and flexibility exercises three times a week for eight weeks during chemotherapy. The control group received no specific information regarding exercise. The outcome measures including body composition, muscle strength, balance, flexibility, cardiovascular fitness and health-related quality of life (HRQoL) were assessed at baseline and eight weeks following baseline. Results: The body composition (body fat percentage, *p* = 0.002; skeletal muscle percentage, *p* = 0.008), dynamic balance (*p* = 0.01), muscle strength (upper extremity, *p* = 0.037; lower extremity, *p* = 0.025) and HRQoL (*p* = 0.001) showed a significant difference between the exercise group and the control group eight weeks following baseline. Significant deteriorations were noted in flexibility, muscle strength, cardiovascular fitness and several domains of HRQoL scale in the control group at eight weeks following baseline. Conclusions: This study found that a combined aerobic, resistance and flexibility exercise program during chemotherapy may improve physical fitness (i.e., muscle strength, balance, flexibility and body composition) and HRQoL and alleviate the deterioration of cardiovascular fitness in patients with HNC. Further research studies with large sample sizes are warranted to investigate the long-term effects of exercise in this population.

## 1. Introduction

More than 650,000 people are diagnosed with head and neck cancer (HNC) every year worldwide. The main anti-cancer treatments for HNC include chemotherapy, radiotherapy, surgery, or a combination. However, these anti-cancer treatments may cause a number of side effects, such as weight loss, fatigue, nausea, prolonged bed rest, impaired functional performance, reduced muscle strength, sleep quality disorder and decreased health-related quality of life (HRQoL) during and after treatment [1,2,3,4]. Chemotherapy and radiotherapy have been shown to be associated with low levels of physical activity [5].

Cisplatin is one of the commonly used chemotherapeutic drugs for the treatment of HNC. The possible mechanism of anticancer action of cisplatin is associated with its ability to interact with DNA, denature the double helix, inhibit the repair of DNA and induce apoptosis in tumor cells [6,7]. Despite the positive anticancer effects of cisplatin, various toxic side effects including nausea/vomiting, nephrotoxicity, ototoxicity, peripheral neuropathy, increased liver enzymes, myelosuppresion and immunosuppression are associated with cisplatin treatment [6,8]. In addition, previous animal studies also reported that cisplatin administration may induce muscle atrophy [9,10]. However, to date, only animal studies have been conducted to test the effectiveness of exercise interventions in diminishing side effects (e.g., acute kidney injury [11,12] and muscle wasting [13]) of cisplatin treatment.

Exercise has been recommended by the American Cancer Society Head and Neck Cancer Survivorship Care Guideline as effective in reducing fatigue and improving functional capacity and HRQoL [14]. Previous studies have also shown that exercise may have a beneficial effect on pain, physical function, fatigue and HRQoL in patients with HNC who have completed cancer treatments [15,16,17], and that exercise is safe and feasible in patients who are undergoing concurrent chemoradiotherapy [18,19,20,21,22] or radiotherapy [23,24,25]. A systematic review by Bye et al. [26] investigated the effects of physical exercise and nutrition interventions during curative (radiotherapy) treatment for patients with HNC and found that nutrition alone and physical exercise alone may be effective in improving body composition and objective physical function. However, the available evidence in the literature for the effectiveness of exercise in managing physical fitness in patients undergoing only chemotherapy (without radiation) for HNC is limited.

One randomized controlled trial (RCT) has investigated multimodal exercise for body composition, cardiopulmonary fitness and exercise capacity for head and neck cancer patients receiving chemotherapy with some positive findings reported [27]. A single-arm trial also reported significant differences in exercise capacity and oxidative stress after eight weeks combined aerobic and resistance exercise training program in patients receiving chemotherapy for HNC [28]; however, little is known about the effect of exercise on other physical outcomes (e.g., muscle strength, balance, flexibility) in patients with HNC who are undergoing chemotherapy (i.e., cisplatin) only. Therefore, the aim of this study was to determine whether exercise training improves physical fitness in patients receiving chemotherapy for HNC.

## 2. Methods

### 2.1. Study Design

We conducted a single centre, parallel RCT at a tertiary hospital in Tainan, Taiwan. This study was approved by the ethical committee of National Cheng Kung University Hospital Institutional Review Board, Tainan, Taiwan (A-ER-106-137) and registered prospectively in Thai Clinical Trials Registry (TCTR20200701002).

### 2.2. Participants and Procedure

The participants for this study were recruited from the outpatient clinic of Hematology and Oncology of Department of National Cheng Kung University Hospital (NCKUH), Tainan, Taiwan. Inclusion criteria included: (1) adults (≥20 years old), (2) a diagnosis of HNC, (3) scheduled to receive chemotherapy, (4) no brain tumor metastasis, (5) no serious complications and (6) no history of mental illness. Exclusion criteria included: (1) being unable to provide consent, (2) pregnant or lactating women, (3) neurological disorders (e.g., stroke), (4) musculoskeletal disorders that limited mobility (e.g., myopathy, amputation), (5) severe psychiatric disorders (e.g., bipolar disorder and schizophrenia), (6) signs of severe organ failure and (7) estimated survival time less than six months. After the written informed consent and baseline data were obtained from the participants, participants were assigned randomly to “exercise” or “control” groups. One of the investigators generated a list of allocation sequences by using a computer-generated code, and the allocation was concealed. The collection of data in this study was not blinded. To reduce ascertainment bias, we conducted specific awareness training to avoid bias of researchers. We performed a “case manager” system for investigation and data collection (a single person undertook all assessments and a single person undertook exercise training). If cancer recurrence or progression was found during the study period, participants were free to cease the participation at any time.

### 2.3. Intervention

**Figure 1:**
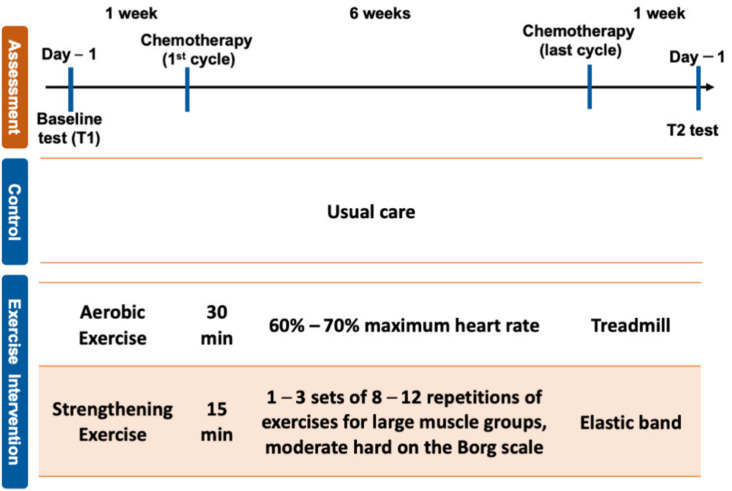
Study intervention protocol and time points of outcome measurements 1.

The exercise group received the intervention three days before the first cycle of chemotherapy. The chemotherapeutic drug, cisplatin, was given for four weeks, and the exercise program was carried out during chemotherapy and continued for four weeks after completion of the first cycle of chemotherapy. The dosage of cisplatin was 100 mg/m^2^, cisplatin was injected by IV on day 1 and repeated 6 weeks. Total duration of intervention was eight weeks, and each session was undertaken at the gym in National Cheng Kung University. The exercise program consisted of moderate-intensity aerobic, resistance and flexibility exercises supervised by a physical therapist and was provided three times a week. Each session lasted 90 min and included 5-min warm-up and a 5-min cool-down exercises. The training intensity of aerobic exercise on a treadmill was 60–70% maximum heart rate (HR). The resistance exercise using Thera-Band or free weight consisted of 1–3 sets of 8–12 repetitions of exercises for large muscle groups of the upper and lower body and the muscles in the core of the body. The intensity of resistance exercise was a rating of perceived exertion (RPE) of moderate hard on the Borg scale. The flexibility exercise consisted of static stretching of large muscle groups, including shoulder extensors and flexors, elbow extensors and flexors, hip extensor and flexors and knee extensors and flexors. The intensity, volume and frequency of the exercise program were increased when the participant was willing and able to progress. Participants in the control group received no specific information regarding exercise and were given general education including information about the side effects of chemotherapy. Figure 1 presents the protocol of this study and time points of outcome measurements.

### 2.4. Outcome Measures

Socio-demographic and medical characteristics including age, gender, tumor site and cancer stage were recorded at baseline. Body composition, muscle strength, balance, flexibility, cardiovascular fitness and HRQoL were assessed at Time 1 (baseline) and Time 2 (8 weeks following baseline).

### 2.5. Body Composition and Muscular Strength

Body composition, body weight and body mass index (BMI) were measured using a body composition monitor (OMRON Karada Scan 214, Osaka, Japan). Upper extremity (U/E) strength was assessed using the 30 s arm curl test [29] by which the number of bicep curls completed within 30 s while maintaining the weight of the hand (male: 3 kg, female: 2 kg) was recorded. A 30 s chair stand test [30] was used to assess lower extremity (L/E) strength. The participants sat in the middle of the chair (placed against a wall) with back straight, laid their feet flat on the floor, and crossed their arms at the wrists and held against the chest. On a verbal signal, participants stood up and then returned to the seated position. Participants were encouraged to complete as many performances as possible within 30 s, and the number of times the participants stood up in 30 s was recorded [31].

### 2.6. Balance

Time up and go (TUG) test [32] was performed according to the procedures originally described by Mathias et al. [33]. The time required to stand up from a sitting position, walk 3 m, turn around, walk back to the chair and return to the sitting position was recorded in seconds.

### 2.7. Flexibility

Upper-extremity flexibility was assessed using the back scratch test [29]. The distance between the middle fingertips when one arm reached behind the back and the other reached over the shoulder in an attempt to reach the middle finger of the other hand was measured and recorded in centimeters. The chair sit-and-reach test [29] was used to assess L/E flexibility. While in a sitting position on a chair, the participant extended one leg forward with the knee straight and heel on the floor. The ankle was bent at 90°. The participant placed one hand on top of the other and then slowly bent forward from the hip until the fingers touched the toes. The distance between the finger tips and the tip of toes was measured and recorded in centimeters.

### 2.8. Cardiovascular Fitness

The 3 min step test [34,35] was used to measure cardiovascular fitness. Participants were asked to step on and off a 30 cm high bench for three minutes. At the end of three minutes, the participant stopped immediately and sat down on the stair. The heart rate (HR) in beats per minute was recorded at 1–1.5 min, 2–2.5 min and 3–3.5 min. In addition, blood pressure (assessed with a sphygmomanometer), oxygen saturation (assessed with an oximeter), rate of perceived exertion (assessed with the Borg Rating of Perceived Exertion scale [36]), time of completion and physical fitness index ([(time of completion) × 100/(1–1.5 min + 2–2.5 min + 3–3.5 min) × 2]) were measured and recorded.

### 2.9. Health-Related Quality of Life (HRQoL)

Health-related quality of life was measured with the European Organization for Research and Treatment of Cancer Quality of Life Core Questionnaire (EORTC QLQ-C30) [37] and the head and neck module (EORTC-QLQ-H&N35) [38]. All scale/single-item measures ranged in score from 0–100. A high score on the functional and the global QoL scale represents a high (better) level of functioning and high HRQoL, while a high score on the symptom scale represents a high (worse) level of symptomatology.

### 2.10. Statistical Analysis and Sample Size Calculation

Sample size calculation was conducted using the G*Power software (version 3.1.0). Based on the results from a previous study investigating the effect of a multidimensional exercise intervention on physical capacity, well-being and quality of life in cancer patients undergoing chemotherapy [39], which showed an effect size of 0.465 for the maximum oxygen uptake (VO_2_ max) after intervention, a sample of 30 participants per group would allow us to detect a difference between the exercise group and the control group, with a power of 80% and an α level of 0.05.

Data were analyzed on the as-treated (AT) basis. Descriptive data were indicated as mean and standard deviations, and number and percentage. The baseline data were compared between the control and exercise groups using the independent *t*-test and chi-squared test. The changes in outcomes between two groups across time were assessed with ANOVA test with significance difference level set at 0.05. All data analyses were done using SPSS software version 22.0 (Ins., Chicago, IL, USA).

## 3. Results

### 3.1. Demographic and Medical Characteristics

**Figure 2:**
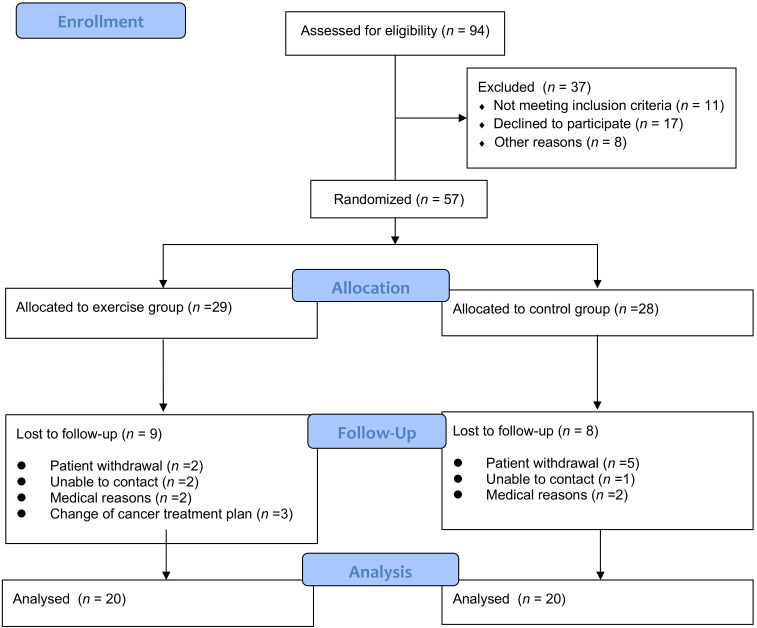
Recruitment of subjects.

A total of 57 participants were recruited from September 2015 to May 2017, and 40 completed the follow-up assessment (Figure 2). The demographic and medical characteristics of the participants are shown in Table 1. The majority of participants had stage 1–2 (*n* = 23, 57.5%) disease. Half of the participants had a diagnosis of nasopharyngeal cancer (*n* = 20). There was no significant difference in participant demographic and medical characteristics between the control and exercise groups. This exercise program was supervised by physical therapists, and the compliance of the exercise group was 93.1%.

### 3.2. Body Composition

Table 2 reports data on body composition. Significant differences were found in body fat percentage (*p* = 0.002) and skeletal muscle percentage (*p* = 0.008) between the exercise and control groups after intervention.

### 3.3. Balance, Flexibility and Muscle Strength

In the exercise group, a significant difference was found in dynamic balance (*p* = 0.01) between two groups post-intervention (Table 3). The flexibility of U/E (*p* = 0.018) and L/E (*p* = 0.028) was significantly improved in the exercise group after intervention. After eight weeks, the control group had a significant decrease in the flexibility of U/E. 

In addition, the muscle strength of U/E (*p* = 0.037) and L/E (*p* = 0.025) were significantly improved in the exercise group compared with the control group after eight weeks intervention. The control group showed a significant decrease in the muscle strength of L/E (*p* = 0.013) after eight weeks (Table 3).

### 3.4. Cardiovascular Fitness

There were no significant changes in resting HR and peak HR between and within two groups (Table 4). However, the physical fitness index decreased significantly in the control group from Time 1 to Time 2 (*p* = 0.031). Significant differences were found between two groups in heart rate recovery after 3 min step test. 

### 3.5. HRQoL

After eight weeks intervention, significant between-group differences were found in several domains of EORTC QLQ-C30 and H&N35 scores (global health status, physical functioning, role functioning, emotional functioning, fatigue, appetite loss, feeling ill and weight gain) (Table 5).

## 4. Discussion

This is the first RCT reporting the effect of an 8-week exercise training program on muscle strength, flexibility, dynamic balance and several other cardiovascular fitness endpoints, as well as impact on body composition and HRQoL in patients undergoing chemotherapy for HNC. Significant between-group differences were observed in body fat percentage, skeletal muscle percentage, dynamic balance, U/E and L/E muscle strength and HRQoL between the exercise group and the control group over the 8 weeks intervention period. Moreover, significant deteriorations were noted in U/E flexibility, L/E muscle strength, physical fitness index, heart rate recovery and several domains of HRQoL scale in the control group. These results demonstrate that a combined aerobic, resistance and flexibility exercise program may improve physical fitness (i.e., muscle strength, balance, flexibility and body composition) and HRQoL and attenuate the deterioration of cardiovascular fitness during chemotherapy for HNC.

The findings of greater improvements in body composition, muscle strength and HRQoL in the exercise group compared with the control group concur with the results from previous RCTs of combined aerobic and resistance exercise in patients with HNC undergoing concurrent chemoradiotherapy [22], chemotherapy [27] and radiotherapy [24,40]. In Zhao et al.’s research, patients undergoing concurrent chemoradiotherapy for HNC were randomized into a maintaining physical activity during cancer treatment (MPACT) group or a control group [22]; the study found a significant beneficial effect of knee strength, mental health and HRQoL in the MPACT group, who received a 14-week functional resistance and walking program, compared to the control group. Yen et al. [27] reported that an 8-week combined aerobic exercise and resistance exercise intervention significantly improved body composition and exercise responses in patients undergoing chemotherapy for HNC as compared with a sedentary group. Our results on HRQoL, in particular fatigue, are in line with previous studies in patients receiving radiotherapy for HNC [40,41]. Moreover, we also found significant differences in U/E and L/E flexibility and dynamic balance within the exercise group and between two groups, respectively. These findings were unable to be compared with previous studies due to differences in study populations, intervention program and outcome measures used.

Gait imbalance is one of the main complaints of patients suffering from chemotherapy-induced peripheral neuropathy (CIPN) [42]. A systematic review [43] reported that only one study showed significant improvements in dynamic balance control after aerobic endurance training, sensorimotor training and strength training in patients with lymphoma [44]. Our finding adds further evidence on the benefits of exercise on dynamic balance in patients receiving chemotherapy for HNC. Given that chemotherapy and its side effects including problems with balance are associated with falls in cancer survivors [45], future RCTs should investigate the effects of exercise on prevention of falls in patients undergoing chemotherapy for HNC. HNC survivors often complain of limited shoulder range of motion due to anti-cancer therapy which is associated with reduced HRQoL [46,47]. According to the American College of Sports Medicine Physical Activity Guidelines for Cancer Survivors [48], cancer survivors should stretch major muscle groups on days that other activities are performed. In addition, flexibility exercise has also been reported as one of the most popular exercise types among HNC survivors [49]. While this study noted significant improvements in U/E and L/E flexibility over the 8-week intervention, Jansen et al. reported that a 12-week self-help exercise program consisting of flexibility for head, neck and shoulders, range-of motion and lymphedema exercises, and a self-care education program were not effective in improving shoulder disability [50]. However, Jansen et al. did not include flexibility as an outcome measure. Future research is needed to confirm the effects of combined aerobic, resistance and flexibility training program on flexibility in patients with HNC.

A limitation of this study was the lack of usage of the cardiopulmonary exercise testing, a gold-standard test to determine cardiopulmonary fitness levels [51]. Nevertheless, we used the 3-min step test that has been shown to have a significant correlation with the maximum oxygen uptake (V0_2_ max) [52]. The pilot nature, small sample size, high drop-out rate (29.8%) and lack of long-term follow ups are other limitations of the study. This pilot RCT is underpowered because the sample size was calculated based on the VO_2_ max data from the previous similar study [39]. As the first RCT on this topic, the findings of this study may provide data for more accurate sample size calculations in future trials. Moreover, the heterogeneity of the surgical techniques and the multicenter nature of the study decreased the credibility of the results. Moreover, the inclusion of participants undergoing cisplatin only means that these findings may not be generalized to patients receiving other types of chemotherapy. Although the study cohort included patients with tumors at different sites (including tongue, oropharyngeal, parotid and nasopharyngeal), nasopharyngeal cancer is generally regarded as separate from other cancers of the head and neck in its etiology, clinical behavior and treatment [53], hence the findings of this study may not be applicable to all HNCs. In addition, both groups contained slightly more early-stage (1–2) tumors (57.5%); therefore, findings need to be interpreted with caution due to the possible impact of cancer stage on symptom burden [54]. As a combined aerobic, resistance and flexibility exercise training program was provided in this study, it was not possible to determine which exercise components or a combination attributed to the positive intervention effects.

Current clinical practice guidelines [14,55] on exercise for patients with HNC focus on management of patients after HNC treatment and provide no specific recommendations for maintaining or improving physical fitness and HRQoL in patients undergoing adjuvant treatment for HNC as evidence is currently scarce. The findings from this pilot RCT suggest that clinicians should consider offering moderate-intensity exercise to patients receiving HNC chemotherapy based on tumor site and cancer stage.

## 5. Conclusions

In summary, this study suggested that an eight-week combined aerobic, resistance, and flexibility exercise training program may improve physical fitness including body composition, balance, muscle strength and HRQoL and alleviate the deterioration of cardiovascular fitness in patients with HNC. Further research studies with larger sample sizes are warranted to investigate the long-term effects of exercise in this population. The results of this study provide valuable evidence to the current literature and suggest that health care professionals working with patients with HNC should encourage patients to participate in supervised exercise early during chemotherapy.

## Figures and Tables

**Table 1:** Demographic and medical characteristics of the exercise group and control group.

Clinical Characteristic	Control (*n* = 20)	Exercise (*n* = 20)	*p*-Value
Age	54.3 (9.9)	52.1 (15.7)	0.655
Gender (M/F)	11/9	14/6	
Tumor site, *n* (%)			0.532
Tongue	3	3	
Nasopharyngeal	9	11	
Oropharyngeal	6	6	
parotid	2	0	
Cancer stage, *n* (%)			0.749
Stage 1–2	11	12	
Stage 3–4	9	8	

Data were presented as Mean (SD).

**Table 2:** Body composition between the exercise group and control group at pretest and posttest.

Body Composition	Group	Time 1 Mean (SD)	Time 2 Mean (SD)	*p* (Within Group, T1–T2)	*p* (Between Group, T1)	*p* (Between Group, T2)
Body weight (kg)	Control (*n* = 20)	61.6 (12.1)	61.9 (12.4)	0.865	0.916	0.916
	Exercise (*n* = 20)	60.6 (11.4)	59.9 (11.0)	0.310		
BMI (kg/m^2^)	Control (*n* = 20)	23.1 (3.2)	23.2 (3.3)	0.842	0.158	0.139
	Exercise (*n* = 20)	21.2 (3.4)	21.1 (3.3)	0.866		
Body fat percentage	Control (*n* = 20)	25.9 (3.5)	25.8 (2.5)	0.495	0.114	0.002 *
	Exercise (*n* = 20)	25.5 (4)	21.0 (2.8)	0.348		
Visceral fat level	Control (*n* = 20)	8.9 (5.1)	8.9 (4.9)	0.803	0.415	0.357
	Exercise (*n* = 20)	6.9 (4.8)	6.7 (4.8)	0.317		
Skeletal muscle percentage	Control (*n* = 20)	31.5 (2.9)	31.4 (2.4)	0.865	0.067	0.008 *
	Exercise (*n* = 20)	34.1 (3.4)	34.5 (2.4) *	0.612		

* *p* < 0.05. BMI, Body mass index. Data were presented in mean (SD).

**Table 3:** Balance, flexibility and muscular strength outcomes between the exercise group and control group at pretest and posttest.

Physical Fitness	Group	Time 1 Mean (SD)	Time 2 Mean (SD)	*p* (Within Group, T1–T2)	*p* (Between Group, T1)	*p* (Between Group, T2)
Dynamic balance (s)	Control (*n* = 20)	8.3 (1.29)	8.4 (1.29)	0.705	0.184	0.01 *
	Exercise (*n* = 20)	7.29 (2.21)	6.42 (1.51)	0.196		
**Flexibility (cm)**						
Upper extremity	Control (*n* = 20)	−9.2 (14.4)	−12.3 (16.6)	0.011 *	0.359	0.832
	Exercise (*n* = 20)	−19.6 (16.7)	−11 (15.6)	0.018 *		
Lower extremity	Control (*n* = 20)	4.0 (10.8)	0.2 (11.6)	0.191	0.275	0.944
	Exercise (*n* = 20)	−0.92 (5.1)	4.28 (4.19) *	0.028 *		
**Strength (reps/30 s)**						
Upper extremity	Control (*n* = 20)	23.4 (8.4)	21.06 (5.38)	0.093	0.672	0.037 *
	Exercise (*n* = 20)	24.1 (6.3)	27.0 (5.8)	0.027 *		
Lower extremity	Control (*n* = 20)	15.6 (4.37)	13.1 3(3.87)	0.013 *	0.158	0.025 *
	Exercise (*n* = 20)	19.7(6.58)	20.14 (7.04)	0.752		

* *p* < 0.05; Data were presented in mean (SD).

**Table 4:** Cardiovascular fitness and heart rate recovery between the exercise group and the control group at pretest and posttest.

3 min Step Test	Group	Time 1 Mean (SD)	Time 2 Mean (SD)	*p* (Within Group, T1–T2)	*p* (Between Group, T1)	*p* (Between Group, T2)
**Heart Rate (HR)**						
Rest (bpm)	Control (*n* = 20)	77.9 (16.4)	79.0 (16.8)	0.461	0.084	0.888
	Exercise (*n* = 20)	94.3 (16.3)	79.1 (4.3)	0.051		
Peak (bpm)	Control (*n* = 20)	126.6 (23.4)	109.4 (23.6)	0.109	0.191	0.177
	Exercise (*n* = 20)	130.3 (29.9)	118.3 (15.9)	0.310		
**Time of completion (s)**	Control (*n* = 20)	167.1 (38.7)	165.4 (28.9)	0.345	0.857	0.186
	Exercise (*n* = 20)	163.3 (30.3)	163.3 (17.8)	0.818		
**Physical fitness index**	Control (*n* = 20)	78.0 (25.1)	67.6 (19.8)	0.031 *	0.053	0.503
	Exercise (*n* = 20)	56.7 (10.1)	64.7 (25.1)	0.237		
**Heart rate recovery**						
1–1.5 min (beats)	Control (*n* = 20)	41.7 (6.4)	44.9 (8.3)	0.132	0.007 *	0.168
	Exercise (*n* = 20)	52.9 (5.7)	48.9 (8.6)	0.237		
2–2.5 min (beats)	Control (*n* = 20)	36.6 (6.6)	41.7 (9.6)	0.028 *	0.002 *	0.305
	Exercise (*n* = 20)	46.7 (4.2)	44.3 (5.6)	0.344		
3–3.5 min (beats)	Control (*n* = 20)	33.7 (7.8)	40.6 (8.6)	0.003 *	0.005 *	0.621
	Exercise (*n* = 20)	44.4 (5.2)	41.9 (4.9)	0.237		

* *p* < 0.05; Data were presented in mean (SD). HR, Heart Rate.

**Table 5:** Health-related quality of life between the exercise group and control group at pretest and posttest.

Domain	Group	Time 1 Mean (SD)	Time 2 Mean (SD)	*p* (Within Group, T1–T2)	*p* (Between Group, T1)	*p* (Between Group, T2)
**EORTC QLQ-C30**						
Global health status	Control (*n* = 20)	5.75 (0.55)	5.45 (1.31)	0.379	0.792	0.001 *
	Exercise (*n* = 20)	5.85 (1.60)	7.10 (1.48) *	0.014		
Physical functioning	Control (*n* = 20)	6.15 (0.93)	9.50 (3.15)	<0.001 *	0.745	0.018 *
	Exercise (*n* = 20)	6.05 (1.00)	7.45 (1.93)	0.001		
Role functioning	Control (*n* = 20)	2.60 (0.82)	4.65 (1.39)	<0.001 *	0.357	0.024 *
	Exercise (*n* = 20)	2.85 (0.87)	3.65 (1.30)	0.053		
Emotional functioning	Control (*n* = 20)	6.15 (1.93)	10.50 (3.29)	<0.001 *	0.295	<0.001 *
	Exercise (*n* = 20)	5.45 (1.19)	7.35 (1.95)	0.003 *		
Cognitive functioning	Control (*n* = 20)	2.55 (0.69)	2.70 (1.08)	0.624	0.484	0.121
	Exercise (*n* = 20)	2.70 (0.66)	3.30 (1.30)	0.069		
Social functioning	Control (*n* = 20)	3.50 (0.83)	5.70 (1.38)	<0.001 *	0.664	0.139
	Exercise (*n* = 20)	3.60 (0.60)	5.10 (1.11)	<0.001 *		
Fatigue	Control (*n* = 20)	3.70 (0.67)	7.95 (1.88)	<0.001 *	0.272	<0.001 *
	Exercise (*n* = 20)	3.95 (0.76)	5.85 (1.78)	<0.001 *		
Nausea and vomiting	Control (*n* = 20)	2.30 (0.47)	5.50 (1.79)	<0.001 *	0.744	0.087
	Exercise (*n* = 20)	2.35 (0.49)	4.60 (1.43)	<0.001 *		
Pain	Control (*n* = 20)	2.55 (0.60)	3.15 (1.42)	0.090	0.575	0.330
	Exercise (*n* = 20)	2.45 (0.51)	2.80 (0.70)	0.050 *		
Dyspnea	Control (*n* = 20)	1.30 (0.47)	1.70 (0.98)	0.088	0.5204	0.719
	Exercise (*n* = 20)	1.40 (0.50)	1.60 (0.75)	0.297		
Insomnia	Control (*n* = 20)	1.25 (0.44)	1.55 (0.83)	0.111	0.690	0.732
	Exercise (*n* = 20)	1.30 (0.47)	1.65 (0.75)	0.110		
Appetite loss	Control (*n* = 20)	1.55 (0.89)	3.30 (0.86)	<0.001 *	0.687	0.012*
	Exercise (*n* = 20)	1.15 (0.36)	2.55 (0.95)	<0.001 *		
Constipation	Control (*n* = 20)	1.30 (0.47)	2.15 (0.81)	0.037*	0.526	0.151
	Exercise (*n* = 20)	1.45 (0.51)	1.80 (0.70)	0.130		
Diarrhea	Control (*n* = 20)	1.15 (0.37)	1.55 (0.89)	0.056	0.052	0.600
	Exercise (*n* = 20)	1.35 (0.48)	1.70 (0.86)	0.190		
Financial difficulties	Control (*n* = 20)	2.15 (2.49)	2.05 (0.83)	0.428	0.258	0.852
	Exercise (*n* = 20)	1.95 (0.60)	2.10 (0.85)	0.527		
**QLQ-H&N35**						
Oral pain	Control (*n* = 20)	4.85 (0.93)	5.95 (2.35)	0.051	0.298	0.815
	Exercise (*n* = 20)	5.20 (1.15)	6.10 (1.61)	0.082		
Swallowing problems	Control (*n* = 20)	4.60 (0.68)	4.75 (0.71)	0.179	0.183	0.523
	Exercise (*n* = 20)	4.90 (0.72)	4.60 (0.75)	0.137		
Sense problems	Control (*n* = 20)	2.35 (0.58)	4.10 (1.37)	<0.001 *	0.295	0.072
	Exercise (*n* = 20)	2.55 (0.60)	3.35 (1.18)	0.095		
Speech problem	Control (*n* = 20)	4.05 (1.14)	5.05 (1.82)	0.049 *	0.298	0.918
	Exercise (*n* = 20)	4.40 (0.94)	5.00 (1.17)	0.048 *		
Social eating problems	Control (*n* = 20)	4.65 (0.87)	6.55 (1.93)	<0.001 *	0.154	0.794
	Exercise (*n* = 20)	5.10 (1.07)	6.40 (1.66)	0.001		
Social contact problems	Control (*n* = 20)	6.75 (1.25)	6.95 (2.68)	0.793	0.667	0.823
	Exercise (*n* = 20)	6.90 (0.91)	6.80 (1.32)	0.793		
Sex problems	Control (*n* = 20)	3.65 (1.03)	4.10 (1.44)	0.304	0.013*	0.085
	Exercise (*n* = 20)	4.35 (0.59)	4.85 (1.22)	0.126		
Teeth problems	Control (*n* = 20)	1.30 (0.47)	1.35 (0.48)	0.716	0.478	0.569
	Exercise (*n* = 20)	1.20 (0.41)	1.45 (0.60)	0.204		
Mouth opening problems	Control (*n* = 20)	1.1 (0.30)	1.40 (0.82)	0.083	0.222	0.657
	Exercise (*n* = 20)	1.25 (0.44)	1.30 (0.57)	0.789		
Dry mouth	Control (*n* = 20)	1.25 (0.44)	1.50 (0.88)	0.330	0.503	0.836
	Exercise (*n* = 20)	1.35 (0.48)	1.45 (0.60)	0.606		
Sticky saliva	Control (*n* = 20)	1.30 (0.47)	1.65 (1.03)	0.201	0.268	0.730
	Exercise (*n* = 20)	1.15 (0.36)	1.55 (0.75)	0.072		
Coughing	Control (*n* = 20)	1.25 (0.44)	1.50 (0.82)	0.096	0.503	0.664
	Exercise (*n* = 20)	1.35 (0.48)	1.60 (0.59)	0.204		
Feeling ill	Control (*n* = 20)	1.40 (0.50)	2.75 (0.91)	<0.001 *	0.176	<0.001 *
	Exercise (*n* = 20)	1.20 (0.41)	1.75 (0.64)	0.002 *		
Pain killers	Control (*n* = 20)	1.25 (0.44)	1.50 (0.51)	0.056	0.324	0.107
	Exercise (*n* = 20)	1.40 (0.50)	1.25 (0.44)	0.330		
Nutritional supplements	Control (*n* = 20)	1.5 (0.512)	1.75 (0.44)	0.171	0.532	0.054
	Exercise (*n* = 20)	1.40 (0.50)	1.45 (0.51)	0.748		
Feeding tube	Control (*n* = 20)	1.00 (0.00)	1.00 (0.00)	-	-	-
	Exercise (*n* = 20)	1.00 (0.00)	1.00 (0.00)	-		
Weight loss	Control (*n* = 20)	1.40 (0.503)	1.55 (0.51)	0.267	0.324	0.214
	Exercise (*n* = 20)	1.25 (0.44)	1.35 (0.49)	0.428		
Weight gain	Control (*n* = 20)	1.05 (0.223)	1.10 (0.31)	0.577	0.324	0.044 *
	Exercise (*n* = 20)	1.00 (0.00)	1.45 (0.68)	0.008 *		

* *p* < 0.05; Data were presented in mean (SD).
